# Supplementary figures and images for: Pharmacogenetic allele variant frequencies: An analysis of the VA’s Million Veteran Program (MVP) as a representation of the diversity in US population
Source: PLoS One. 2023 Feb 24;18(2):e0274339. doi: 10.1371/journal.pone.0274339 (PMC9956596; doi:10.1371/journal.pone.0274339)

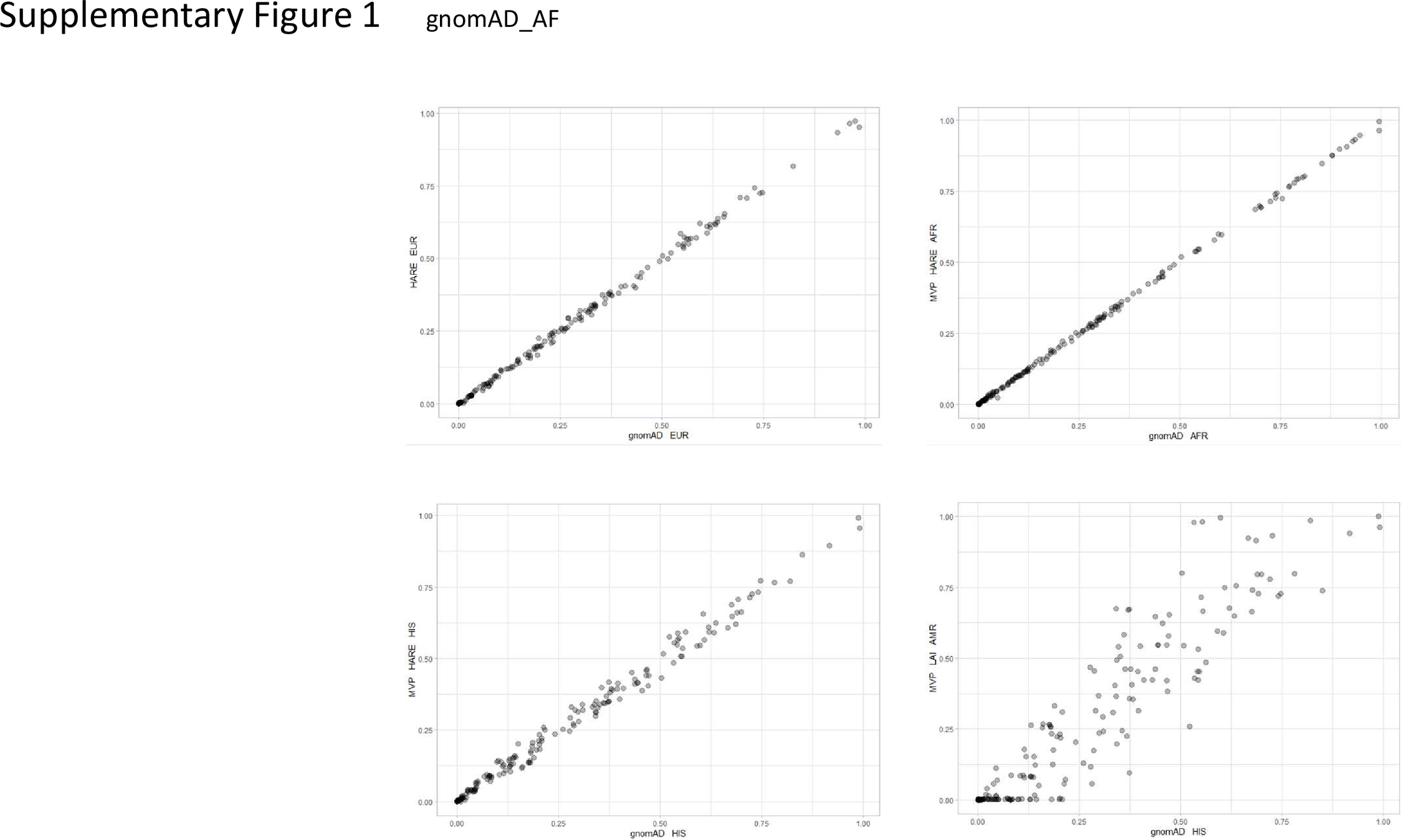

Supplement: S1 Fig — For all three, correlation with gnomAD allele frequencies is high (R2>0.99). In the lower right we compare allele frequencies for gnomAD HIS and Local Ancestry Inference (LAI) derived allele frequencies for the AMR track of the HARE HIS group (R2 = 0.91). We use three-way local ancestry deconvolution (EUR, AFR, AMR). (TIF) [file pone.0274339.s001.tif]

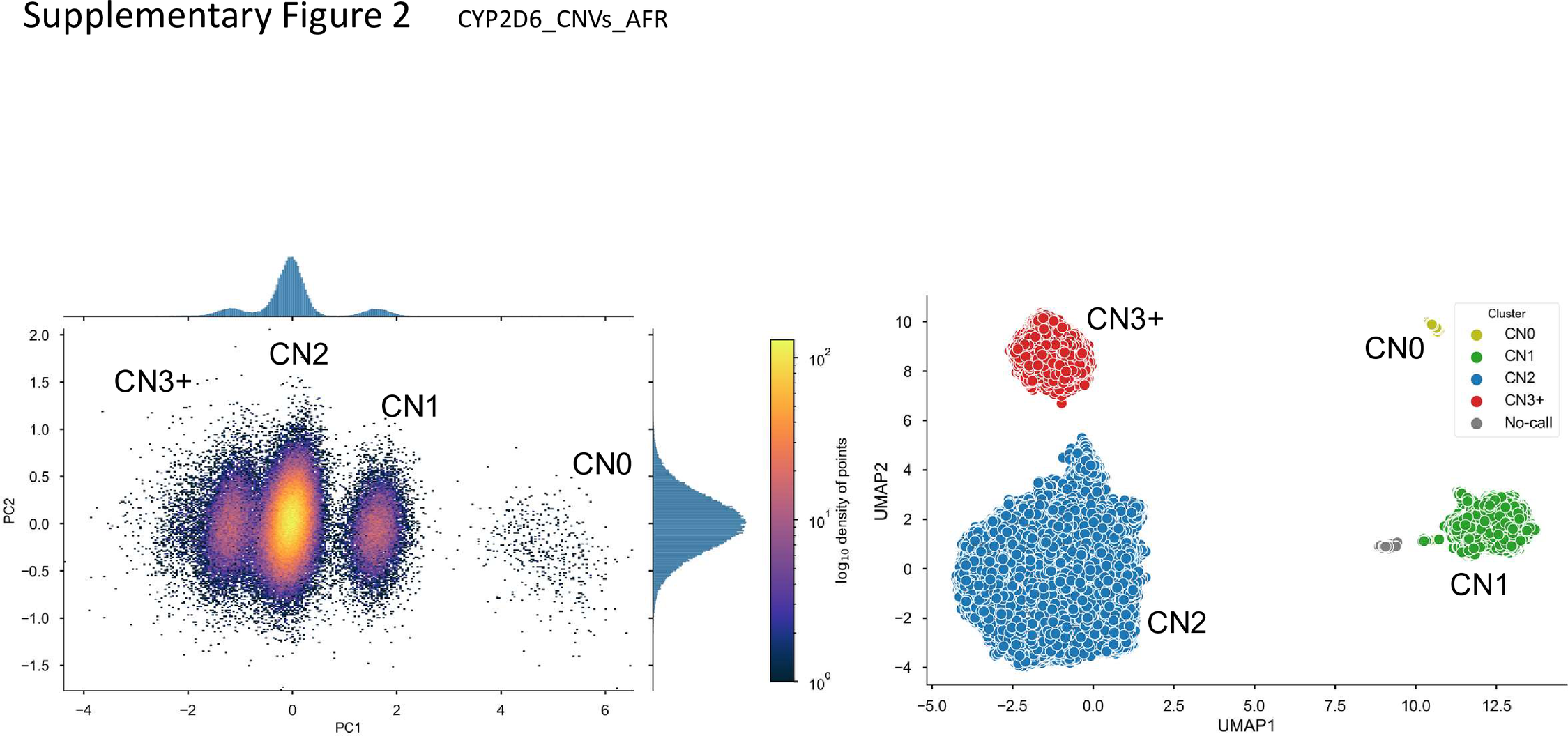

Supplement: S2 Fig — Results are shown just for the HARE AFR cohort; clusters were derived using (a) Principal Components Analysis (PCA) and (b) UMAP(13). UMAP significantly reduces assignment ambiguity. (TIF) [file pone.0274339.s002.tif]
