# Supplementary material for: Pharmacogenetic allele variant frequencies: An analysis of the VA’s Million Veteran Program (MVP) as a representation of the diversity in US population
Source: PLoS One. 2023 Feb 24;18(2):e0274339. doi: 10.1371/journal.pone.0274339 (PMC9956596; doi:10.1371/journal.pone.0274339)
Supplement: Attachment — Submitted filename: Response to reviewers 2022-12-12.docx [file pone.0274339.s004.docx]

Boston, December 12, 2022

______________________________________________________________________________

**RESPONSE TO REVIEWERS**

**Reviewer 1**

Reviewer #1: The authors analyzed the frequencies of pharmacogenetics relevant variants in a large US population. Regarding the frequencies of these variants, it is not clear whether those not mentioned in the manuscript are publicly available?
 **Reply:** Allele frequencies for HARE groups and LAI tracks, are provided as a supplementary table with this submission.

Other issues:
What is the message that the authors try to convey by conducting the LAI analysis?
**Reply:** This is a very helpful comment. We now realize that we did not clarify the benefits of LAI despite mentioning its usage in multiple places in the manuscript. We substantially revised the discussion to that effect. We repeat the argument here while referencing the relevant sections in the original manuscript (first submission).

LAI conveys important information on allele frequency distribution in under-represented populations. As noted in lines 72-75 allele frequency is an important consideration in the formulation of clinical genotyping guidelines provided by groups such as CPIC and DPWG. As noted in lines 124-131, the AMR track is a much better sampling of the AMR genome as it exists today in the US population compared to the small, and not necessarily representative, number of samples from AMR populations (60 vs 15,000). Another example is provided in lines 172-183. We present the allele frequency of single and double-copy deletions of *CYP2D6.* The variant frequency is low, especially for double deletions. Therefore, it is inappropriate to derive it from reference panel, even under the assumption that the reference panel is representative of the general US population. Finally, as noted in lines 225-231, our LAI work “underscores the need for individual typing rather than reliance on self-reported ethnicity on drug delivery decisions in clinical practice”. We hope this manuscript promotes the adoption of personalized medicine in under-represented populations.

The presentation of Fig.1 is neither clear nor informative, the authors should try to present in another way or just highlight some of the genes.
**Reply:** We agree that figure 1 is not useful for understanding the allele frequency of individual variants or even genomic regions. Such figures are common in population genetics manuscripts as they convey an impression of variation scale and genome-wide distribution. With the editor’s and reviewer’s consent, we would like to keep Figure 1 as it currently exists. A more detailed picture is offered in Figure 2, where we focus on *CYP2D6*. For practical use in research and development of clinical guidelines we expect researchers to consult the supplementary table included with resubmission of the manuscript.

Why the column ‘LAI’ of ‘EUR’ in the table of Fig.2 is identical to ‘MVP HARE’, and why some of the SNVs do not have a value in LAI? I suggest the authors to switch the X and Y axis of the Fig.2.
**Reply:** As the reviewer notes, the ‘LAI’ of ‘EUR’ is identical to ‘MVP HARE’. The reason is that we did not run LAI in the ‘MVP HARE’ sample. On average the EUR track contributes >99% of the genome in the ‘MVP HARE’ cohort and LAI does not have an impact of derived allele frequencies. Thus, the two allele frequencies are identical by default. We kept the column on the table for uniformity of presentation. We should have noted this in the Figure legend. We are grateful to the reviewer for pointing this out. We modified the figure legend.

We estimate imputation quality per site and ethnicity. We do not present allele frequencies for poorly imputed sites. It was an oversight, and we are grateful to the reviewer for pointing this out. We updated the figure legend.

Regarding the figure orientation, we originally created the plot with X and Y axis reversed. However, that made it difficult to align the figure with the table. We defer to the editor on whether the entire figure (table+plot) should be rotated for final publication.

In line 156, the authors referred Fig.2., does it mean that the SNVs in Fig.2 belong to the ones that in the CYP2D6*5 CNV region?
**Reply:** The SNVs are within the CNV region. This creates a dilemma. For individuals harboring a single copy we can count just one allele, or we can treat them as homozygotes (two copies). Here we follow convention and treat them as homozygotes.

In line 182, it should be ‘fewer 2-copy samples’ rather than ‘fewer diploid samples’.
**Reply:** The proposed wording clarifies the statement; updated as suggested.

In Supplementary Fig.1, it is better to show the correlation coefficient and the P-value.

**Reply:** Added R^2^ as suggested.

**Reviewer 2**

Reviewer #2: Overall, this is a well written manuscript and will add important information about pharmacogene allele frequencies in US populations.

Mostly, I have minor comments.
Genes should be in italics in accordance with HGVS nomenclature.
 **Reply:** Corrected
Please make sure that all abbreviations/acronyms are written out for clarity of the reader. Many are documented below.
 **Reply:** Corrected throughout manuscript
line 32 -I prefer the term variants over polymorphisms unless all variants that are being discussed are greater than 1% frequency.
 **Reply:** We believe that in the context of this sentence the term polymorphisms is appropriate. The vast majority of variants of interest have allele frequencies > 1%.
Line 88- first use of HARE, please write out acronym.
 **Reply:** Corrected as suggested
line 139 - while the use of the rsID is acceptable, for PGx readers, please also include *allele.
 **Reply:** Modified as suggested
line 143 - please confirm if the drug name need to be capitalized.
 **Reply:** Corrected, thanks for catching this.
line 144 - while the use of the rsID is acceptable, for PGx readers, please also include *allele.
 **Reply:** Modified as suggested
line 153 - b-blockers, either write out beta or use Greek symbol as appropriate for publisher.
 **Reply:** changed to beta-blockers
line 160 - first use of PCA, please write out acronym.
 **Reply:** Modified as suggested
lines 181-182 - remove word clearly (that is up to the reader to determine) and "a lot"; make sure sentence reads well after revision.
 **Reply:** Modified as suggested
lines 193, 393 - prefer the term variants to SNPs
 **Reply:** Here we use SNVs to highlight the different classes of variants: single point mutations, copy number variants and HLA haplotypes/alleles. We think that using SNVs highlights the distinction and we would prefer to keep the current formulation.
Line 194 - first use of HIBAG, please write out acronym.
 **Reply:** Updated as suggested.
Line 199 - first use of SCAR, please write out acronym.
 **Reply:** Updated
Line 199 - first use of SJS/TEN, please write out acronyms.
 **Reply:** Updated and reformulated sentence.
lines 206-207 - Please delete "something we hope to address in the future". If needed this would be for the discussion as a future research.
 **Reply:** Deleted as suggested
line 370 - QC'd is lab lingo, please write out
 **Reply:** Corrected as suggested
Line 374 - SHAPEIT appears to be some program, is this an acronym? Is there a source that needs to be cited?
 **Reply:** We provided the hyperlink to the software web site.
lines 375, 397 - Minimac4 appears to be some program, is this an acronym? Is there a source that needs to be cited?
 **Reply:** We provided the hyperlink to the software web site.
Lines 416-420 - check font. I am not sure that matters for formatting for publication.
 **Reply:** Corrected
